# Eight‐Year Experience of Hedgehog Pathway Inhibitors at Three Tertiary Referral Centres in the Australian State of Victoria

**DOI:** 10.1111/ajd.14544

**Published:** 2025-06-10

**Authors:** Christian Gan, Nicholas Manuelpillai, Peter Foley, Christopher McCormack, Michelle S. Goh

**Affiliations:** ^1^ Skin Health Institute Carlton Victoria Australia; ^2^ Department of Dermatology St Vincent's Hospital Melbourne Fitzroy Victoria Australia; ^3^ The University of Melbourne Parkville Victoria Australia; ^4^ Department of Surgical Oncology (Dermatology), Peter MacCallum Cancer Centre Melbourne Victoria Australia

**Keywords:** basal cell carcinoma, basosquamous carcinoma, Gorlin syndrome, Hedgehog pathway inhibitors, sonic hedgehog inhibitors, sonidegib, vismodegib

## Abstract

This retrospective analysis of Hedgehog inhibitor treatment in 32 patients with Gorlin syndrome, locally advanced and metastatic basal cell carcinoma (BCC) at three tertiary referral centres in Victoria, Australia from April 2017 until 30 June 2024 demonstrated an 84% overall objective response rate (partial and complete response combined). However, 90% of patients experienced adverse effects impacting quality of life. Secondary acquired drug resistance occurred in 77% (10/13) of locally advanced and metastatic BCC patients after a median duration of 13 months. Further work is needed to optimise the neoadjuvant use of Hedgehog inhibitors with radiotherapy or surgery given poor long‐term Hedgehog inhibitor tolerability and to develop strategies to counteract the issue of acquired resistance.

## 1 Introduction

Vismodegib and sonidegib are Hedgehog pathway inhibitors (HHI) available as systemic treatments for adult patients with metastatic or locally advanced basal cell carcinomas (BCCs) who are not amenable to curative surgical resection and/or radiation therapy.

A recent Australian single centre retrospective analysis of 23 patients from the state of New South Wales outlining real‐world efficacy and safety of HHI has been recently published [1]. We complement this in presenting 8 years of experience of HHI at three institutions that represent the vast majority of advanced public dermatologic oncology care in the state of Victoria, Australia, with retrospective data on 32 patients from the time of Pharmaceutical Benefits Scheme (PBS) listing in April 2017 until 30 June 2024. All patients were managed by multidisciplinary teams consisting of dermatology, surgical, medical and radiation oncology.

## 2 Results and Discussion

Table 1 presents demographic and treatment data, including BCC subcategory indication for HHI. Six patients (19%) had the high risk and therapeutically challenging basosquamous carcinoma tumour subtype. Median follow‐up duration (Table 2) was 51 months, but 5 patients (4 with Gorlin) transitioned to PBS from the Vismodegib STEVIE trial [2], and 1 patient (Gorlin) from the Sonidegib BOLT trial [3], hence follow‐up for these 6 patients was up to 12 years.

**TABLE 1:** Baseline demographics, category of basal cell carcinoma indication for Hedgehog inhibitor treatment and Hedgehog inhibitor drug.

	Patients, *n* = 32
Age—median; mean age (range), years	65.5; 63 (33–90)
Gender—female: male (%)	9 (28%): 23 (72%)
Category of BCC indication for treatment	
Gorlin Syndrome	13 (41%)
Non‐Gorlin Syndrome	19 (59%)
Numerous BCCs^a^	2 (6%)
Locally advanced BCCs	10 (31%)
Numerous + locally advanced BCCs^a^	2 (6%)
Locally advanced + metastatic BCCs	3 (9%)
Metastatic BCCs	2 (6%)
Hedgehog inhibitor drug	
Vismodegib^b^	25 (78%)
Sonidegib^b^	17 (53%)

^a^ Numerous BCCs: *n* = 4 patients who had at least 50 previous lifetime BCCs and 10 or more BCCs at the time of commencement of Hedgehog inhibitor.

^b^ 15 patients received vismodegib only, 9 received vismodegib followed by sonidegib, 1 received sonidegib followed by vismodegib, 7 received sonidegib only.

**TABLE 2:** Duration of treatment, dose, duration of response, follow‐up and deaths. The median drug dose compared to continuous once daily dosing for each Hedgehog inhibitor is shown.

	Patients, *n* = 32
Duration of Hedgehog inhibitor treatment (mean; 95% confidence interval), months	
Vismodegib	40; 29–69
Sonidegib	24; 5–43
Vismodegib and sonidegib combined	39; 25–63
Dose (% of continuous once daily dosing) (median; range)^a^	
Vismodegib	63%; 27–100
Sonidegib	75%; 34–100
Duration of response to first‐line Hedgehog inhibitor in locally advanced and metastatic BCC (median; range), months^b^	
Vismodegib (*n* = 10)	13; 2–25
Sonidegib (*n* = 3)	6; 5–20
Follow‐up duration (median; range), months	51; 2–146
Deaths	8
Related to BCC	4
Unrelated to BCC	4

^a^ Dose % compared to full continuous daily dosing calculated according to prescription & medication dispensing against total number of days.

^b^ Duration of response: defined as the time interval between the time of earliest partial or complete response to the time of progressive disease or death or last follow‐up.

The choice of vismodegib versus sonidegib was at the prescriber's discretion, taking into account indication for HHI (e.g., metastatic BCC), comorbidities (e.g., medications affecting cytochrome P450 3A4), also noting that sonidegib was PBS‐listed 1 year after vismodegib. There was no difference in efficacy between vismodegib and sonidegib.

Our cohort's overall response rate to HHI is comparable to published real‐world data [1, 4]: 84% objective response rate (ORR), 75% partial response (PR), 9% complete response (CR) (Table 3).

**TABLE 3:** Best response to Hedgehog inhibitor as per RECIST v1.1 response criteria by clinical ± radiological imaging [5].

Best response	Gorlin (*n* = 13)	nBCC (*n* = 2)	laBCC (*n* = 10)	nBCC + laBCC (*n* = 2)	laBCC + mBCC (*n* = 3)	mBCC (*n* = 2)	All laBCC^a^ (*n* = 15)	All mBCC^b^ (*n* = 5)	Overall
Complete response	1	0	2	0	0	0	2	0	3 (9%)
Partial response	11	2	5	1	3	2	9	5	24 (75%)
Stable disease	0	0	2	0	0	0	2	0	2 (6%)
Progressive disease	0	0	1	1	0	0	2	0	2 (6%)
Lost to follow up	1	0	0	0	0	0	0	0	1 (3%)

Abbreviations: laBCC, locally advanced basal cell carcinomas; mBCC, metastatic basal cell carcinomas; nBCC, numerous basal cell carcinomas.

^a^ ‘All laBCC’ are all cases of laBCC combined.

^b^ ‘All mBCC’ are all cases of mBCC combined.

Our cohort's complete response (CR) rates are lower than other published data [1, 4, 6]. Our CR definition was determined by a sustained CR until the last follow‐up, regardless of whether on active treatment or drug holiday, rather than assessment of outcome within a particular active treatment cycle. Additionally, amongst the locally advanced BCCs (laBCC) and metastatic BCCs (mBCC) in our cohort, there consisted a notable number of patients (*n* = 10) with complex skin cancers (BCCs and SCCs) that had been resistant or recurred after previous extensive surgery ± radiation ± systemic therapy (conventional cytotoxic, targeted epidermal growth factor receptor inhibitors, checkpoint inhibitor immunotherapy) pre‐HHI.

Of 3 cases who achieved CR: 1 had Gorlin syndrome and remained clinically clear of any BCCs on sonidegib; 1 had laBCC who tolerated near 100% sonidegib dosing for 20 months and achieved clinical and radiological clearance of extensive mid‐face disease that was sustained until death from unrelated causes; and 1 with upper and lower eyelid laBCC who had 5 months' neoadjuvant vismodegib followed by consolidative radiotherapy, remaining clinically clear 9 months post‐radiation.

### 2.1 Gorlin Syndrome

Our cohort had a comparatively larger proportion of patients with Gorlin syndrome—of 13 patients, 2 patients abandoned treatment within 2 months because of adverse effects (one had demonstrated PR prior to drug cessation; one did not have assessment of response due to being lost to follow‐up) The 11 patients who continued treatment had remarkable and sustained clinical suppression of BCCs (10 PR, 1 CR) for as long as they remained on HHI, with no or very few additional surgical procedures required, compared to pre‐HHI when dozens of BCCs per year required treatment. All patients with Gorlin required dose reduction to achieve a balance between adverse effects and BCC control (median dose: 57% of continuous once daily dose, range 33–73; calculated by prescription and medication dispensing against total number of days). When patients underwent treatment breaks, BCCs were noted to clinically recur as erythematous macules at the same sites; hence, 10/11 Gorlin patients were assessed as PR.

### 2.2 Treatment‐Emergent Acquired Drug Resistance, Squamous Differentiation in BCC, New Keratinocyte Cancers

Of the 15 patients with laBCC, 9 had initial PR. However, 8 of these 9 cases of PR had subsequent resistance: this secondary progressive disease (PD) occurred after a median duration of 13 months (range 5–24 months). Three of these 8 had mBCC in addition to laBCC; the acquired resistance occurred in laBCC lesion(s) rather than mBCC lesion(s).

In the 2 patients with mBCC alone (without concurrent laBCC), secondary resistance after initial PR also occurred in both (occurring after 2 and 25 months respectively).

Both the 2 patients with numerous BCCs (without concurrent laBCC) had PR to vismodegib, with marked reduction in the number of BCCs for the follow‐up duration of 135 and 49 months respectively.

Two patients had vismodegib treatment‐emergent squamous differentiation of the target BCCs (after 5 and 24 months respectively). These were histologically confirmed BCCs prior to vismodegib, and rebiopsy upon tumour progression showed SCC. Given that the treatment‐emergent SCCs arose within the same sites of the target BCCs, it is unlikely that these were concomitant new primary SCCs.

Seven patients had treatment‐emergent new primary keratinocyte cancers at separate sites (2 patients had BCCs, 3 had SCCs, 2 had BCCs and SCCs), reflecting the severe field dysplasia in our patient population and the presence of dysregulated mechanisms other than the Hedgehog pathway alone.

There was no correlation between dose reduction and treatment‐emergent resistance, squamous differentiation, or new primary keratinocyte cancers. For instance, despite near 100% adherence to vismodegib daily dosing in 4 patients, 1 patient with laBCC developed SCC in the eyelid BCC at 24 months, 2 patients with laBCC plus mBCC developed secondary resistance at 8 and 12 months, and 1 patient with mBCC developed new primary aggressive SCC at 25 months that was treated with cemiplimab and failed to regain vismodegib response on rechallenge after 5 months—the latter 3 patients were high‐risk basosquamous carcinoma phenotypes.

### 2.3 General Lack of Response to Switching HHI

The attempt to switch to the alternate HHI in 7 patients with primary or acquired resistance was not successful in 6/7. One patient regained a short duration of HHI response for 3 months (first‐line vismodegib PR for 14 months was followed by treatment‐emergent resistance, second‐line cemiplimab for 11 months, third‐line sonidegib resulting in PR for 3 months and then PD after).

Upon failure of HHI, 8 patients proceeded to the next line of cemiplimab immunotherapy (with subsequent 3 PR and 5 PD to cemiplimab).

### 2.4 HHI Adverse Effects, Dosing

Consistent with published data, most patients (90%) reported quality of life impact from treatment‐emergent adverse effects (TEAEs), predominantly muscle spasms, alopecia, dysgeusia, fatigue and weight loss (Table 4).

**TABLE 4:** Treatment‐associated adverse events graded by severity.

	Grade 0 (%)	Grade 1 (%)	Grade 2 (%)	Grade 3 (%)	Grade 1–3 (%)
Alopecia	10 (33.3%)	4 (13.3%)	16 (53.3%)	0 (0%)	20 (66.7%)
Muscle spasms	3 (10%)	8 (26.7%)	19 (63.3%)	0 (0%)	27 (90%)
Dysgeusia	8 (26.7%)	8 (26.7%)	14 (46.7%)	0 (0%)	22 (73.3%)
Weight loss	14 (46.7%)	6 (20%)	4 (13.3%)	6 (20%)	16 (53.3%)
Anorexia	12 (40%)	9 (30%)	9 (30%)	0 (0%)	18 (60%)
Fatigue	13 (43.3%)	6 (20%)	11 (36.7%)	0 (0%)	17 (56.7%)
Nausea	24 (80%)	4 (13.3%)	2 (6.7%)	0 (0%)	6 (20%)
Diarrhoea	21 (70%)	7 (23.3%)	2 (6.7%)	0 (0%)	9 (30%)

*Note:* The severity of adverse events graded using the 2017 Common Terminology Criteria for Adverse Events (CTCAE) version 5.0 https://ctep.cancer.gov/protocoldevelopment/electronic_applications/docs/ctcae_v5_quick_reference_5x7.pdf. 2 patients abandoned treatment at 2 months due to adverse effects, but the adverse effect was not specified. Both were excluded from this table. Percentages are thus calculated out of 30 patients. Muscle Spasms: G1 mild pain; G2 moderate pain, limiting instrumental activities of daily living (ADL); G3 severe pain, limiting self‐care ADL; Alopecia: G1 < 50% of normal, obvious only on close inspection; G2 ≥ 50% loss for the individual, readily apparent to others, for which a hair piece is necessary if desired for camouflage. Weight loss: G1 (< 10% from baseline), G2 (10%–20%), G3 (> 20%). Anorexia & Nausea: G1 without alteration in eating habits; G2 oral intake altered without significant weight loss. Fatigue: G1 relieved by rest; G2 not relieved by rest, limiting instrumental ADL; G3 not relieved by rest limiting self‐care ADL. Diarrhoea: G1 increase of stool < 4 per day over baseline; G2 increase of 4–6 stools per day.

Abbreviations: ADL, activities of daily living; G, grade.

Six patients (19%) abandoned HHI treatment within 4 months or less due to intolerance. Of the 26 patients who remained on HHIs beyond 4 months, 18 patients (69%) required dose reductions or treatment breaks to tolerate ongoing HHI. The primary reasons stated for dose reduction or discontinuation were muscle spasms for 18 patients, alopecia for 2 and adverse effect unspecified for 4.

Upon recommendation in the latter 4 years of our cohort, 12 patients tried prophylactic Magnesium and L‐Carnitine supplements to prevent and/or reduce the severity of muscle spasms: 9 and 3 patients continued Magnesium and L‐Carnitine, respectively.

Interestingly, 4 patients (2 vismodegib, 2 sonidegib) reported no or minimal muscle spasms. One of these patients who switched HHI because of primary resistance had found no muscle spasms or dysgeusia to initial sonidegib (21 months) but reported both muscle spasms and dysgeusia to subsequent vismodegib, with onset of muscle spasms within 4 weeks of starting vismodegib. Three other patients tried a switch to the alternate HHI because of intolerance of adverse effects, but found no difference in the severity of adverse effects.

Weight loss appeared to be significant in our cohort, occurring in 16 patients (53%, all grades) and Grade 3 severity (> 20% loss from baseline weight) occurring in 6 patients (20%).

All patients were initiated on continuous daily dosing until the adverse effects became problematic. After this, patients usually self‐determined the dosing regimen that worked best for them—the various regimens included 1–2 months on and off, alternate day, every 3–4‐day dosing, 5 out of 7‐day dosing, and a few patients had several months' breaks before resuming HHI.

## 3 Limitations and Future Research

Limitations include the small size of this cohort and non‐randomised drug selection (including participation in single agent clinical trials). There was large variability in treatment breaks and dose reduction regimens amongst patients.

Further research is needed to optimise HHI tolerability to facilitate ongoing therapy (especially in Gorlin syndrome) and to address acquired resistance which has limited the durability of response in laBCC and mBCC. The strategy of neoadjuvant short‐term HHI followed by radiotherapy or surgery is gaining traction [7, 8]. It is hoped that combination treatments (including with immunotherapy and future novel drugs targeting alternative or downstream effector pathways) can also be effective strategies against HHI resistance [9].

## 4 Conclusion

In combination with the recent publication on 23 patients in New South Wales, we now have real‐world data on HHI efficacy and safety for a total of 55 patients in Australia [1].

Concordant with literature to date, major challenges in HHI therapy remain—poor tolerability of adverse effects and treatment‐emergent acquired drug resistance. It can be difficult balancing the quality of life impact of HHI against tumour control in this often elderly and comorbid population presenting with advanced BCCs who have failed or are not suitable for standard surgical or radiation treatment. There appears to be no difference between vismodegib and sonidegib in their efficacy and tolerability profiles [10]. Switching between drugs to improve tolerability or efficacy is useful in the rare instance.

## Disclosure

Copyright Declaration: One patient in this manuscript has previously been published as a case report in 2021: Mansour KP, O'Duffy F, Webb A, Goh M, Morrison E. About Face: Can Vismodegib change the Treatment Paradigm of Locally Advanced Basal Cell Carcinoma? *ANZ J Surg*. 2021 Jun;91 (6):1304–1306. doi: 10.1111/ans.16399

## Ethics Statement

Peter MacCallum Cancer Centre Human Research Ethics Committee (HREC/69197/PMCC) approval.

## Conflicts of Interest

C.G., N.M., M.G. declare no conflicts of interest. A/Prof Peter Foley: investigator vismodegib STEVIE trial, advisory board, consultant, research grant, speaker, travel grant Sun Pharma; advisory board, investigator, research grant, speaker Novartis. A/Prof Christopher McCormack is an Editorial Board member of Australasian Journal of Dermatology and a co‐author of this article. To minimise bias, they were excluded from all editorial decision‐making related to the acceptance of this article for publication.

## Data Availability

Individual patient case data are not made publicly available due to potential compromise of privacy. Supporting data are available on request from the corresponding author.
